# Efficacy pilot study of the DSM-5 Cultural Formulation Interview in a specialized mental healthcare inpatient unit for adolescents in Norway

**DOI:** 10.3389/fpsyt.2025.1595131

**Published:** 2026-01-02

**Authors:** Nina Therese Øversveen Svamo, Valerie DeMarinis, Sigrid Helene Kjørven Haug

**Affiliations:** 1 Research Center for Existential Health, Innlandet Hospital Trust, Faculty of Social and Health Sciences, University of Inland Norway, Lillehammer, Norway; 2 Research Center for Existential Health, Innlandet Hospital Trust, Brumunddal, Norway; 3 Department of Public Health and Clinical Medicine, Umeå University, Umeå, Västerbotten, Sweden; 4 Research Center for Existential Health, Innlandet Hospital Trust, Faculty of Social and Health Sciences, University of Inland Norway, Elverum, Norway

**Keywords:** adolescents, clinicians, communication, Cultural Formulation Interview, person-centered care, psychiatry

## Abstract

**Background:**

The DSM-5 core Cultural Formulation Interview (CFI) is designed with the agenda of letting the patient’s perspective become as important for treatment and care as the clinician’s assessments. This is in line with the person-centered turn in every part of the health care system in Norway. However, international CFI research on majority populations and on adolescents remains scarce. This study is the first to test the feasibility, acceptability, and clinical utility of core CFI with adolescents in a specialized mental healthcare inpatient unit in Norway.

**Methods:**

The study used a mixed methods design with three stages, inspired by and expanding the CFI testing research in the United States: 1) Cultural analysis of the clinical context and CFI training, 2) Data gathering with CFI interviews, debriefing instruments, and semi-structured interviews with six consecutive adolescents (aged 14–17 years) with various severe mental health problems, and multi-method data gathering with three interdisciplinary CFI-trained clinicians, and 3) Efficacy evaluation of the CFI.

**Results:**

Adolescents and clinicians reported positive perceptions of the CFI’s feasibility, acceptability, and clinical utility. The CFI supported service user involvement and treatment planning, consistent with international evidence. Both patients and clinicians described the CFI as a type of intervention that initiated a process of reflection and deeper understanding of challenges as well as resources, with patients expecting their narratives to be understood, shared, and integrated into treatment planning.

**Conclusion:**

This study contributes to person-centered care (PCC) research by underscoring the importance of actively involving adolescents in their treatment processes. Based on these findings, the clinic initiated a request to evaluate the integration of the CFI process (core interview and use of this information in treatment planning) into standard treatment protocols through an implementation study, which is currently underway.

## 1 Introduction

The introduction is divided into three sections leading up to the aim of this research study.

### 1.1 Person-centered Care and Adolescents

Person-centered care (PCC) in general healthcare and in mental healthcare in particular has seen a substantial increase in the last 20 years (1–3). Guidelines of the World Health Organization (WHO) make it clear that the provision of PCC is a human right in mental healthcare settings (3). Recent research has found that PCC may offer several benefits over standard care (2). These benefits concern rates of adherence and self-management, resulting in improved cost-effectiveness, service satisfaction, and quality of life (2). Morgan and Yoder (1) analyzed the concept of PCC in the context of an inpatient post-acute healthcare environment and identified four defining attributes as a foundation for practice: holistic, individualized, respectful, and empowering. However, despite increasing interest in PCC, there is a lack of clarity among clinicians about what PCC is and how it is to be practiced (1, 4).

Communication skills constitute a pivotal component of PCC (4). In a scoping review, Constand and colleagues (5) identified effective communication as one of three components of a PCC approach, across different frameworks and models. The two other components were partnership and health promotion. Effective communication includes the following: sharing information, active listening, compassionate and empowering care, respect for patient autonomy, and sensitivity to patients’ needs. The studies revealed that prioritizing effective communication strategies improves outcomes when integrated into health services (5). There are studies suggesting that more attention should be directed towards the communication skills of clinicians working with adolescents in mental healthcare (6, 7). A British study addressing the views of adolescents aged 11–18 who received mental healthcare found that communication skills were more relevant than actual therapeutic approaches (8). A recent scoping review of adolescents’ voices on self-engagement in mental health treatment, found that being listened to and met with kindness were central factors for self-engagement in the therapeutic process (9).

Receiving direct responses from adolescents and paying attention to the unique expectations and feelings they bring to treatment are essential factors in successful treatment (9–12). This may increase understanding of how they perceive mental health problems (13). Their view of what therapy involves may differ from clinicians’ perceptions (12). In a recent systematic review on patient involvement in adolescent mental healthcare by Viksveen et al. (14), adolescents expressed a desire for active engagement in their treatment processes. However, a systematic review of factors influencing PCC in child and adolescent mental healthcare (15) found that adolescents are seldom active participants in their treatment. These reviews recommend a greater focus on the unique needs of each person and more knowledge about the best ways in which adolescents want to and can be involved in their treatment, suggesting that healthcare for adolescents should become more person-centered (14, 15).

### 1.2 The Cultural Formulation Interview

The Cultural Formulation Interview (CFI) (16) from DSM-5 (17, 18) is a useful and validated tool to elaborate patients’ perspectives on dealing with illness and health in relation to their cultural concerns. The Outline for Cultural Formulation (OCF) from the DSM-IV (19) provided a framework for assessing the cultural features of an individual’s mental health problem and how these relate to the patient’s social and cultural context and history (20). The OCF was revised from the DSM-IV original version to the DSM-5 version and again revised for the DSM-5-TR (17, 18). To operationalize the OCF for clinical practice, the DSM-5 included the CFI to provide specific questions for patients to obtain culturally relevant information that could be used in aiding diagnosis and treatment planning processes. The CFI is based on a PCC approach and designed with what often may appear as a radical agenda: “to let the patient’s perspective become as important for treatment and care as the clinician’s assessments” (16). The CFI consists of the core CFI of 16 questions for patient interviewing, and the CFI-Informant Version that can be administrated to close associates of the patient and 12 supplemental modules aimed at particular populations or situations (21). Dixon and colleagues (22) point out the importance of the person’s own culture, background and goals in treatment planning. They recommend the use of the CFI, stating that it is essential “to provide culturally competent and person-centered care”.

The international field trials were a series of studies conducted to test the 14-item pilot version of the CFI for DSM-5 (23). These trials involved 75 clinicians, 318 adult patients from outpatient clinics, mostly with mental health diagnoses, and 86 relatives, to study feasibility, acceptability, and clinical utility across six countries (Canada, India, Kenya, The Netherlands, Peru, and the United States) (23). A mixed-methods evaluation of the field trial data found positive responses to the CFI’s perceived feasibility, acceptability, and clinical utility for both clinicians and patients (23). As there was a need for more specific guidance in regard to implementation, training, and application the results from the international field trials (23) led to a revised 16-item version of the CFI (referred to as the core CFI) in the DSM-5 (17, 18).

Rohlof et al. in the Netherlands (24) evaluated the 14-item version of the CFI and found it to be feasible, acceptable, and useful in psychiatric practice for patients and clinicians, particularly benefiting patients where communication and diagnostic challenges were anticipated. Diaz and colleagues (25) analyzed CFI data from 30 monolingual Spanish participants in the USA, finding that the interview elicited themes about the importance of building trust, addressing stigma, and restoring family and social ties. This information showed the value of routine CFI use in enhancing cultural responsiveness of care, bringing attention to what matters most for this Hispanic population. Aggarwal et al. (26) analyzed the 14-item version of the CFI with 32 adult patients and debriefing instruments with the 32 clinicians who had conducted the CFI, and found that incorporating the CFI into practice, which informed the later 16-item version, can enhance healthcare communication and therapeutic rapport. Patient satisfaction had risen immediately after CFI use (26). In a comparative, cross national study, Hinton and colleagues (27) evaluated relatives’ views, showing that relatives supported the feasibility, acceptability, and clinical utility of the 14-item version of the CFI.

In Scandinavia, evaluating the clinical use of the core CFI has been conducted as part of a larger project in an outpatient psychiatric clinic in Sweden by Wallin and colleagues (28–31). A mixed-method RCT study with patients and clinicians (28) found the core CFI to be feasible, acceptable, and clinically useful. In Denmark, the core CFI has primarily been used with migrant populations (32–34). A mixed-methods study by Skammeritz and colleagues (34) found high patient satisfaction with the CFI among migrant patients in a transcultural mental health clinic. Additionally, a qualitative study by Lindberg et al. (33) found that the core CFI facilitated trust-building and engagement in care among migrant patients.

The positive impact of the CFI on clinical communication in the field trials (26) and CFI studies mentioned using the core CFI with 16 questions, has been confirmed and explored in several additional studies. In the United States, Muralidharan and colleagues (35) explored the core CFI with patients with chronic psychosis in an outpatient Veterans Affairs clinic. The patients found the core CFI valuable in terms of validating their experiences, offering therapeutic benefits, and promoting deeper self-awareness and understanding of their recovery (35). Additionally, the patients described how the clinicians listened and displayed genuine concern for their well-being (35). A case study of a Jamaican American woman with a history of psychosis (36) found that using the core CFI improved communication and helped the clinician understand background diversity. That study underscored the ability of the core CFI to reveal many aspects of the person’s life and context, such as cultural influences (40).

In a clinical synthesis of findings regarding the CFI over the six years since its publication in 2013 to 2018, Jarvis and colleagues (37) found that the core CFI can lead to respectful interaction between patient and clinician and contribute to person-centered healthcare. However, the tool may be more difficult to use with patients with severe symptoms such as psychosis, suicidality, aggression, and cognitive impairment. In a qualitative study in a Mexican setting, where 19 patients were included, the core CFI was found to be important in enhancing communication and building trust (38). The CFI seems to have a positive impact on clinical communication by allowing the clinician to elicit patients’ views on what has caused their symptoms, and by improving the clinician’s ability to care and help patients to access their resources as part of their healthcare solution (39–42). A scoping review on the existing literature on the CFI found that the core CFI was perceived as having a positive effect on medical communication (43), additionally, the core CFI enhanced patient-clinician rapport, facilitated diagnostic and treatment strategies, and promoted in-depth exploration of patients’ illness narratives.

Lewis-Fernández and colleagues (44) published an editorial on a number of articles examining the implementation and impact of work on the CFI from various countries and health settings, indicating that there is room for improvement of the CFI in three key areas: the role of the CFI in transforming standard mental health assessment in psychiatry, identifying strategies to enhance implementation, and improving the CFI instrument from different perspectives. Another qualitative study (45) explored barriers to implementing the CFI, involving 32 patients and 7 clinicians at the New York site included in the DSM-5 international field trial. Eligible patients were of any racial or ethnic background; between 18 and 80 years old. Key barriers reported by patients included: lack of differentiation from other treatments, lack of buy-in, and ambiguity of design. Clinicians highlighted issues such as lack of conceptual relevance, format drift, and redundancy (45). A mixed-methods study conducted by Aggarwal et al. found that training clinicians to use the assessment tool is an important factor in integrating the core CFI in clinical practice (46). The CFI training may be best delivered through a combination of written guidelines, video demonstrations and active behavioral simulations. Research in the United States and Canada (47, 48) found that one hour of training on the core CFI can enhance clinicians’ self-assessed cultural competence and reflection on non-verbal communication skills. The incorporation of the CFI information into electronic health records can be burdensome for providers, particularly when training, time, and organizational incentives are insufficient, leading to superficial documentation or neglect of the CFI’s therapeutic potential (44). A study in the Netherlands (49) evaluated the implementation of the CFI in a large mental health organization and found significant barriers to its adoption, with only 13.2% of intakes utilizing the CFI, noting that provided training in the CFI was not made mandatory for clinicians expected to use the CFI. The study highlighted that clinicians with higher cultural competence were more likely to use the CFI effectively, suggesting that successful implementation may require integrating the CFI into broader cultural competency training (49).

In the qualitative data analyzed by Wallin and colleagues in the mixed-methods RCT study from Sweden (28), some patients found the core CFI questions to be difficult, with abstract questions on identity and background being hard to understand, especially for those with limited language proficiency. This difficulty indicated the need for adjustments to make the questions more comprehensible (28). As part of the larger project in Sweden, a randomized control trial (RCT) showed that the CFI enhanced psychiatric diagnostics by aiding in the identification of depressive disorders and facilitating differential diagnosis among non-native speaking patients (31). Wallin and colleagues conducted two additional studies (29, 30) from the mentioned RCT (31). One study analyzed CFI documentation with non-native Swedish-speaking patients and found that it provided valuable contextualized descriptions of dysfunction, life conditions, and emotions, aiding in identifying psychiatric symptoms despite language barriers (29). The other study focused on native Swedish-speaking patients, revealing themes like distress, social failure, and help-seeking, but suggested a need to reframe questions on cultural identity to improve comprehension and relevance in this group (30). In New York, Aggarwal and colleagues used the core CFI to analyze patient narratives on social stressors, supports, or barriers to care. The study found that social determinants of mental health can be elicited through the core CFI (50).

CFI research on adolescents in mental healthcare has been limited, compared to adult populations (51–55). Both the editorial article by Lewis-Fernández et al. (44) and the literature review by Jarvis et al. (37) underline the inclusion of family systems and engagement with the family in cultural psychiatry as essential. This involves incorporating the entire family into the CFI as a dynamic unit, instead of using an individualistic approach or viewing the CFI as an additional source of information (44). In a RCT from the United States on person-centred approaches among Spanish-speaking families with young children aged 2–7 years, Sanchez and colleagues (53) found that families who received the core CFI had a range of better engagement, including attendance, and treatment outcomes compared to families who received a standard assessment. In addition, the core CFI increased the families’ perception of being understood by the clinician. Building on this study, Sanchez and colleagues (56) applied a culturally informed model of science-based case conceptualization to guide clinicians in using the core CFI in enhancing culturally responsive care for youth and families. This five-step, culturally informed model, builds on the model of Christon et al. (57), and identifies cultural information needed at each step of the therapeutic process, as well as identifying questions from the CFI that can supply this information. The model goes through the entire treatment process from identifying problems through to monitoring and evaluating treatment outcomes.

In a case study by Shem-Tov and colleagues (55), two Ethiopian female adolescents with an apparent eating disorder were included. This case study underlined the significance of employing the CFI to gain a nuanced understanding of symptoms, thereby enabling clinicians to better comprehend the problem in a cultural context. The findings emphasized the critical need for culturally sensitive observation throughout the diagnostic process, especially when addressing mental problems such as eating disorders (55).

### 1.3 Norwegian healthcare for adolescents

In Norway, the National Health and Hospital Plan 2020-2023 (58) highlights that the plan’s overall aim is to create PCC-oriented health services, phrased as the following question to patients and their family members: “What matters to you?”. Involvement of patients in treatment is statutory (59) and is now an indicator in the national quality indicator system (60). The Government’s strategic plan for adolescent mental health (61) emphasizes that improved quality in mental health services will depend on adolescents’ active involvement and influence on treatment decisions. However, reports show that many adolescents with mental health problems in Norway are not receiving the help they need when they need it, and that the legislation and rights for adolescents are not fulfilled as intended (62–65). The plan also specifies that healthcare professionals should take adolescents’ perspectives into greater consideration, which will result in services better adapted to their needs (64).

The CFI was translated into Norwegian in 2015, supported by the Norwegian Directorate of Health (66). The Norwegian translation process of the CFI was accomplished through a number of steps. First, a multi-disciplinary group of healthcare researchers and clinicians, representing each of the four health regions, were provided funding by the Health Directorate to meet on several occasions and begin the translation process. The group also made use of the official Swedish CFI translation as an initial guide and had consultations with Swedish researchers and clinicians using the CFI in Swedish studies. Second, the Norwegian working document, with necessary cultural adaptations was sent to a professional translator, working within the Norwegian healthcare system. Third, two separate back translations of the instrument into English were done by healthcare professionals fluent in both languages. Fourth, protocols for use in making small adjustments or adaptations to the core 16 questions, or the addition of needed sub-questions or clinical explanations, were developed by the Innlandet Hospital Trust CFI research team, notably the second and third authors of this article.

This official Norwegian version, registered with the American Psychiatric Association, was used in the present study. The translation ensures that the CFI aligns with Norwegian language and cultural norms, facilitating its use in clinical practice.

In 2019, the CFI was included in the National Patient Pathway for Specialized Mental Healthcare for Children and Adolescents (67). In this directive, the core CFI is suggested for use as part of the first session in relation to life situation assessments and identification of communication barriers with the use of an interpreter as needed for patients with another cultural background and language (67). While the directive does not explicitly state why the core CFI is recommended so early in the process, this timing can be seen in light of the central goals of the directive, which include increased service user involvement, satisfaction and equitable access to care (67). These goals are parallel to the main purpose of the core CFI (16), which might therefore be a valuable instrument for achieving these ambitions. It is interesting to note that including the core CFI in the first session is also a recommendation from international research (44).

The Norwegian Ministry of Health and Care Services recommends the core CFI as a tool for including the context and perspective of children and young people, especially for minority groups (61). The CFI is highlighted as a means to address the cultural and linguistic background of patients, providing a structured approach to understanding the child’s and young person’s problem, how it is perceived, and for identifying resources within and around the child. This aligns with the Ministry’s broader aim to promote culturally sensitive tools that support the inclusion of children’s and adolescents’ contexts and perspectives in mental healthcare (61).

According to the DSM-5 (24) and elaborated upon by Aggarwal and colleagues (21), the core CFI can be used with all patients in any healthcare setting, regardless of the patients’ cultural and ethnic background. However, most of the research has included patients with minority backgrounds (44), and the clinical settings have primarily been outpatient mental healthcare clinics (43).

Studies have also indicated that the CFI can be valuable even when the clinician and patient share the same ethnic background, as it facilitates an exploration of cultural identity, personal values, and life context beyond ethnicity (28, 30). Research found that native Swedish-speaking patients working with native Swedish-speaking clinicians identified themes such as distress, social failure, and help-seeking, suggesting that the CFI can uncover relevant psychosocial factors even in ethnically homogenous interactions (30).

Further research is needed to explore the use of the core CFI with younger populations (43). Research on the core CFI with ethnic majority populations in general and adolescents in particular has been quite limited. The present study is seen as a contribution to addressing this double gap. To our knowledge, no published studies have used the core CFI in specialized mental healthcare inpatient units for adolescents. This study has been undertaken to begin addressing these gaps in CFI research.

### 1.4 Study aim

The present study aimed to test the efficacy of the core CFI in a specialized mental healthcare inpatient unit for adolescents in Norway. Efficacy was operationalized as feasibility, acceptability, and clinical utility, in line with the CFI international field trials (23).

## 2 Materials and methods

### 2.1 Methodological format

The structured methodological format included documentation and analysis of different stages in the research process in line with the CFI international field trials (23). These included: CFI training with clinicians, a fidelity assessment of clinicians’ administration of the core CFI, interviews with adolescents and clinicians, and debriefing interviews with both groups. The format was further adapted by the third author (VDM), encompassing cultural analysis of the clinical site, and independent efficacy evaluations by clinicians and adolescents. More specifically, this involved interviews with clinicians at different stages of the research process, and interviews with adolescents before completion of treatment. In this way, the core CFI (T1- core CFI) was tested for efficacy at several points (T2, T3) in time during the treatment process. This was described as the CFI process.

**Supplementary Module 9** on “School-Age Children and Adolescents” which covers dimensions such as Cultural Identity, Explanatory Model, Psychosocial Stressors, and Coping and Help Seeking, was not applied in the present study (68). The decision to use the core CFI version and not **Supplementary Module 9** was based on discussions with clinicians and the testing of the core CFI with the adolescent population. See further information in the section below “Participants and setting” and “Group meetings with clinicians and unit manager”.

### 2.2 Participants and setting

Mental health services in Norway are based on the Scandinavian welfare model with the provision of free services to all (69). Mental healthcare is divided into primary (municipal) care and specialist (hospital) care (67). In order for adolescents to be referred to specialist healthcare, their mental health disorders have to be considered as moderate to severe.

This study took place in a specialized mental healthcare inpatient unit for adolescents in Norway. The overall study period spanning from late 2019 through 2024, covered planning, data collection, analysis processes, and feedback to the clinical staff. Patient recruitment took place between May 2020 and February 2021. The inpatient unit provides treatment for adolescents aged 12 to 18, with moderate to severe mental health disorders and a high rate of comorbidity. The unit specializes in treating adolescents with a serious eating disorder. In addition, it welcomes adolescents with various other and often complex difficulties, such as school refusal, depression and self-harm. The inpatient unit has beds for six adolescents and offers treatment for 14 days, six weeks or three months. Adjacent to the inpatient unit, there is a separate school unit. Doctors, psychologists, social workers, nurses, and teachers work together in teams. During the adolescent’s stay, the inpatient unit cooperates with their family, school, local community and the authority that referred them to the unit.

The study’s informants consisted of both clinicians and adolescents. The inclusion criteria for the adolescents were developed in collaboration with the clinicians. This study originally was intended to include adolescents aged 16-18. However, since most of the adolescents were aged 14–16 at inclusion, the core CFI was tested on three adolescents aged 14-15. These adolescents and the clinicians involved both felt that the interview was positive and meaningful, and the age range was therefore adjusted to 14–18 years. Adolescents under the age of 16 were required to provide their own consent as well as parental consent to participate in the study. Following the methodological format of the larger project testing the efficacy of the core CFI and following the CFI process through the treatment process for each patient in the different clinical contexts, six consecutive patients in each clinical context fulfilling the context-specific inclusion criteria and formally consenting to being in the study were included. Therefore, six consecutive adolescent patients were included in this study. Potential participants needed to be assessed as cognitively and emotionally suitable for the core CFI and CFI process by clinicians responsible for the treatment. Gender balance distribution, if possible at the time, was part of the design. Exclusion criteria were: lack of capacity to consent, an ongoing crisis or recent traumatic event, psychosis, severe depression, serious suicide risk, and serious ongoing substance abuse.

The clinicians decided on the time to administer the core CFI in collaboration with the research team. Adjustments to the timing to administer the core CFI were necessitated by challenges in recruiting adolescents, primarily stemming from the situation at the inpatient unit, including factors such as staff illness and COVID-19. The core CFI though intended for administration with each patient as early in the process as possible, was administered at different time points, with two interviews conducted in the first week, one in the second week, two in the third week, and one in the fourth week. This was due to the need to find a suitable time for both the patient and the clinician. Notably, the one in the fourth week was for an adolescent admitted to the clinic for a second time that contributed to the variability in timing, turning the core CFI in this instance to function as a means of reacquainting the clinic staff with the adolescent. During the recruitment period May 2020 to February 2021, a total of eight consecutive adolescents were admitted to the inpatient unit who met the study criteria. Of these, two adolescents initially consented but later withdrew before the CFI interview could be conducted, citing personal challenges and difficulties with the timing of the interview. Finally, six adolescents aged 14–17 years were included. Gender, age, the length of stay, time of conducting the CFI during admission, and diagnostic data from the psychiatric summary in the medical record are provided in **Table 1**. For anonymity, the diagnostic data only indicate the main mental and behavioral disorders. According to medical records, all participants self-identified as ethnic Norwegians.

**Table 1:** Characteristics of the sample.

The core CFI	CFI 1	CFI 2	CFI 3	CFI 4	CFI 5	CFI 6
Patient Gender/Age	F 15	M 17	F 15	F 15	F 14	F 15
Mental and behavioral disorders (ICD 10)	F50.0 Anorexia nervosa	F32.1 Moderate depression episode	F50.0 Anorexia nervosa	F50.0 Anorexia nervosa	F90 Hyperkinetic disorders (ADHD)	F90 Hyperkinetic disorders (ADHD)
The length of stay	Three months	Three months	Three months	Three months	Two months	Three months
Time of conducting the CFI during admission	Fourth week*	First week	Third week	Third week	Second week	First week

*This was in the second treatment period for the patient.

Three clinicians were recruited by the inpatient unit manager: a male specialist psychologist, a female child and adolescent psychiatrist, and a female mental health nurse. The clinicians were ethnic Norwegians. They had considerable experience with child and adolescent mental health services, and had worked in the inpatient unit for five years or more. The CFI interviews were conducted exclusively by the three CFI-trained clinicians. None of them had previous experience with the CFI. Of the six interviews: one clinician conducted three, one clinician two, and one conducted one interview.

### 2.3 Assessment methods during the CFI process

This section provides a detailed explanation of the assessment methods employed to test the efficacy of the CFI process.

#### 2.3.1 CFI training

The CFI process began with a CFI training course for the clinicians. This CFI training course consisted of three elements: a) Preparatory work, b) One-day training session with behavioral simulations, and c) Ongoing consultation and feedback.

a) In the preparatory work, the clinicians watched a video of a core CFI role play (70), tested the CFI on themselves, and analyzed how they were affected by the interview as persons, clinicians and potential patients. The Scandinavian-language video, lasting 38 minutes, was created as a clinical example of how the core CFI can be used. The example presented in the video included a patient with a background in a charismatic church movement, experiencing mental health challenges related to anxiety, depression, and potentially problematic alcohol use. The interviewer was a psychiatrist and an experienced administrator of the CFI. Additionally, each clinician prepared an adolescent case for the behavioral simulation session.

b) One-day training session: The session began with a complete discussion of the video of the CFI interview and of the clinicians’ own testing of the CFI on themselves in their preparatory work.

The adolescent cases prepared by the clinicians were used in behavioral simulation sessions where the clinicians played both interviewer and adolescent. The cases included patients from both majority and minority cultures, and the discussion included cultural analysis of each case. The clinical group discussed each question in the CFI in relation to the patient group. Certain questions and wordings were modified, adjusted and simplified. The importance of probing, asking for examples from daily life, and following the order of the questions from 1–16 were points emphasized by the authors. Both the self-testing analysis and the case session were led by the authors (VDM and SHKH).

c) Ongoing consultation and feedback referred to being available for questions and providing feedback on the CFI process with the six participants throughout their treatment periods. The ongoing consultation and feedback were provided by the authors of this article, who were the project’s principal investigator, project leader and doctoral student.

#### 2.3.2 A cultural formulation interview – fidelity instrument measuring clinicians’ adherence to the core CFI

This instrument was used to assess clinician adherence and competence in the core CFI. The CFI-FI, developed and pilot-tested by Aggarwal and his team (71), was used. The instrument was designed to capture adherence to the CFI method, such as whether the clinicians asked all the 16 questions and followed the specific order as reflected in the core CFI guidelines (71).

#### 2.3.3 Debriefing instruments for patients and clinicians

In line with the CFI field trials (23), the adolescents and the clinicians completed brief questionnaires about their immediate experience of the core CFI: DIP and DIC (23). The DIP and DIC were translated by the authors based on the Swedish version (28). DIP consists of five questions about clinical utility, two questions about feasibility, one question about acceptability and three open questions. DIC has a similar design, with ten questions on clinical utility, four on feasibility, four on acceptability, and two on the instructions given in the core CFI. DIC also contains three open questions.

#### 2.3.4 Semi-structured interviews with adolescents and clinicians

The interview for the clinicians had 14 questions (**Supplementary Material 1**) and was conducted four to seven days after the T1- core CFI, as outlined by Lewis-Fernández and colleagues (23). The clinicians had the opportunity to read through the transcript of the T1- core CFI before the semi-structured interview. The interview assessed the clinicians` perceptions of the most and least helpful aspects of the core CFI related to clinical utility, feasibility and acceptability (23). The clinicians were also asked about their experience of the core CFI in relation to treatment as usual. The first (NTØS) and third (SHKH) authors conducted the interviews.

The adolescents were interviewed twice, using semi-structured interviews (**Supplementary Material 2**). The first interview (T2) was conducted four to seven days after the T1- core CFI (23), and the second interview (T3) during the last week of treatment. The questions in T2 concerned how they experienced the core CFI and the help they received from the inpatient unit. The length of the T2 interview ranged from 2 to 29 minutes. The questions in T3 covered the following main topics: how the treatment had worked, with a particular emphasis on communication/relationship with the therapist/milieu therapists/service user participation and how they had initially experienced the use of the information given in the core CFI. The length of the T3 interview ranged from 4 to 14 minutes. All interviews were conducted face-to-face by the first author (NTØS), except for the first interview, where the second author (SHKH) was also present.

#### 2.3.5 Meetings with clinicians and unit manager

During the CFI process, the authors (NTØS, VDM and SHKH) had four meetings with the clinicians and the unit manager. The first meeting was in the initial phase of the study. The purpose was to get to know the clinicians and the context, and to explore how they understood culture, values, service user participation and meaning. This was part of the cultural analysis of the clinical site, which included an initial inventory of instruments, measures, and process guidelines currently used in the unit regarding diagnosis, treatment planning, and collaboration with other services during or after admission to the unit. The second meeting was halfway through the period, focusing on evaluating and making small adjustments to the CFI process. In addition, the optimal integration of the CFI information for the wider team was discussed. In the third meeting, efficacy was thoroughly reviewed in each of the 18 interviews (6 T1- core CFI, 6 T2 semi-structured interviews and 6 T3 semi-structured interviews) conducted with the six adolescents. This meeting formed the basis for the presentation of the project to the larger clinical group at the unit. The fourth meeting concerned completion of the CFI process at the clinical site.

### 2.4 Instrument analysis

Scoring in DIC and DIP followed the coding system developed by Lewis-Fernandez and colleagues (23). The scale range was as follows: -2 = strongly disagree, -1 = disagree, +1 = agree, and +2 = strongly agree. The item in DIP “Took more time to share my perspective than I wanted” was negatively worded and was therefore reverse scored.

Each item in the Cultural Formulation Interview – Fidelity Instrument (CFI-FI) was scored from 0 to 10 (71). The three authors rated the first T1- core CFI independently, followed by a calibration meeting to clarify questions related to reliability and discuss reasons for variant ratings. A similar procedure was followed for the remaining five T1- core CFI. The independently rated scores were generally similar. Disagreements were discussed until consensus was reached resulting in one consensus score for each item (**Table 2**).

**Table 2:** Results of Cultural Formulation Interview – Fidelity Instrument (CFI-FI) (71).

Item	Mean	Range
*Empathy: Did the clinician paraphrase or name the patient’s emotional state?*	8.3	7-10
*Patient-centeredness: Did the clinician maintain a non-judgmental attitude (not arguing, confronting, or correcting the patient)?*	9.5	7-10
*Clarification: Did the clinician ask follow-up questions to understand unclear patient responses?*	7.6	6-10
*Word matching: Did the clinician use the patient’s preferred illness term whenever the CFI question stem included the term “[PROBLEM]”?*	5.8	4-9
*Illness narration: Did the clinician’s interactions help the patient construct and explore a narrative account of illness or did the clinician seem to rush through the CFI?*	8.3	6-10
*Drift: Did the clinician ask about topics during the CFI session that typically belong to the standard clinical interview (history of present illness, current medications, detailed psychiatric or medical history, family history, social history, mini-mental status examination)?*	10	10
*Order: Did the clinician ask about all topics in order as reflected in the CFI clinician guidelines?*	8.3	8-10
The mean score for all 7 items	8.25	

Each item in the CFI-FI was scored from lowest 0 to highest 10.

### 2.5 Semi-structured interview analysis

During the CFI process, a total of 28 qualitative semi-structured interviews were conducted. The interviews were audio recorded and transcribed verbatim by the first author (NTØS). Transcripts were stored in a password protected database and coded using NVivo for Windows (R1.6), which is a computer-assisted qualitative data analysis software package.

The selected analysis strategy was deductive content analysis as described by Elo and Kyngäs (72). This analysis strategy was chosen due to the central role of the core CFI, serving as both the theoretical foundation and conceptual framework, and the study aim of CFI efficacy. Deductive content analysis was used in coding the adolescent interviews, organized around the main themes outlined in the semi-structured interview guides. In the clinician interviews, we specifically focused on coding and themes most relevant to assessing efficacy. The following analytical steps were applied: selecting the textual unit of analysis, developing a codebook of mutually exclusive categories based on extant theories, coding the data and reporting the data by category (72). Each transcript was first read in order to become familiar with the texts and gain an overall understanding and overview of the content. Key words were noted down along the way. Key words that shared the same meaning were summarized into codes and classified in preliminary categories. Broader categories were identified and finally the themes were refined. Quotes were used to illustrate the participants’ experiences. The authors met to discuss and define emerging themes, review the coding structure, and negotiate discrepancies.

### 2.6 Ethical considerations

The study protocol was approved by the Regional Ethics Committee for Medical Research (REK) in Norway (2019/317-3) and reported to the Data Inspectorate in Innlandet Hospital Trust (case number 109076). All consent forms followed the rules for anonymity, storage of data, and possible advantages and disadvantages that apply to medical research. All participants gave written informed consent. Adolescents under the age of 16 years and their guardians, as noted in the were asked to give their written consent for participation in the study by the responsible clinician at the inpatient unit. In addition to guardian consent, adolescents under 16 years provided consent, ensuring that they understood the study and agreed to participate voluntarily. Adolescents 16 years and older were able to provide consent without additional signed consent from their guardian. Guardians of adolescents older than 16 were informed of their adolescents’ participation. Due to the potentially sensitive nature of the core CFI, the adolescents had available support from their respective responsible clinician after the interview.

## 3 Results

This section presents the findings of the CFI process, addressing the comprehensive efficacy testing of the core CFI. The structure is based on the assessment methods described in the Method section.

### 3.1 Adjustments to the CFI questions on the training day

Composed of 16 questions, the core CFI covers four main domains: definition of the problem, perceptions of cause, context, and support, factors affecting self-coping and past help-seeking, and factors affecting current help-seeking (21). The core CFI is developed to be modified and adjusted by clinicians and researchers to fit the clinical context (28, 42), in this case a specialized mental healthcare inpatient unit for adolescents in Norway. Below is a description of the adjustments and modifications made for use in this context. The entire CFI version used in this context is now published by Svamo and colleagues (73).

To ensure that the words and expressions were suitable for this group of adolescents several changes were introduced in collaboration with the clinicians. These included modification to the introductory text to reassure the adolescents that difficult themes could be followed up later. Norwegian words which were more or less synonymous with the English words were used, rather than more direct translations. For example, the English version asks about the adolescent’s “background”, whereas in Norwegian it is common to ask about *oppvekst* (literally “growing up”), so this word was used to enhance familiarity and comprehension. Four sub-questions were added to the core CFI:

- For question 3 related to “What troubles you most about your problem?”, the sub-question.
- “How do you feel it in your body?” was added because the clinicians expressed the desire to incorporate adolescents’ reflections on bodily experiences, considering this a pivotal and intricate aspect for adolescents.
- For question 8 related to “What are the most important aspects of your background” the sub- questions “What gives you most meaning in life?” and “What makes you experience life as empty or meaningless?” was added because the authors of this article suggested to the clinicians the inclusion of questions related to meaning in life. Mutual agreement was reached to underscore this aspect.
- For question 12 related to “What kinds of treatment, help, advice, or healing have you sought for your [PROBLEM]?” the sub-question “What kind of help have you sought elsewhere, such as at school, from friends, relatives, managers, and social media?” was added because the clinicians want to explore help-seeking from various external sources.

Furthermore, additional contexts/concepts were incorporated to better fit the clinical population:

- For question 6, the word “social media” was added to reflect the role of social media related to support.
- For question 7, the word “school” was added to highlight the potential influence of the school environment related to worsen the problem.
- For question 12, the word “friends” was added as a possible source of help.
- For question 13, the words “bullying”, “challenges in your family” and “various support services that have not understood you” were added to describe factors that may prevent access to needed help.

### 3.2 Cultural Formulation Interview - Fidelity Instrument results

To assess clinician adherence and competence in the CFI, the CFI-FI (71) was used. Each item in the CFI-FI was scored from lowest 0 to highest 10. The CFI-FI results are presented in **Table 2**.

Overall, clinicians administered the core CFI with high fidelity scores. The mean score for all seven items was 8.25. The range varied from 5.8 (*Word Matching*) to 10 (*Drift*). The latter showed that the clinicians covered all the CFI topics, and no other topics, in the T1- core CFI. The item *Patient-centeredness* had the next highest score (9.5). The clinicians did not argue, correct or confront the adolescents. Their attitude was respectful, and the clinicians followed the introduction text stating that there are no right or wrong answers. This also implied exploring words and stories from the adolescents, and moving on to the next question when the adolescents responded that there was nothing more to tell. In one instance the clinician at points corrected the patient’s formulation.

In several of the T1- core CFI, the clinicians provided illustrations of metaphors, such as in question 3, where adolescents expressed uncertainty in identifying the problem in their bodies. Examples of metaphors were “anxiety feeling like a lump in the stomach” and “a tight chest”. If the clinician accepted the adolescent’s responses, acknowledged and confirmed what the adolescent stated and refrained from correction, it resulted in a high score.

For the item *Order*, the range was 8-10. *Order* concerned the extent to which the clinicians followed the structure in the core CFI from question 1 to 16, and asked the entire question, including the introduction to questions 1 and 8. In some CFI, the clinician read the whole question at once instead of dividing it into sequences. The introduction at the beginning of the core CFI and to question 8 were not always read word for word. The item *Clarification* refers to the follow-up questions (questions 1, 4, 12 and 13) in the core CFI, used to understand an unclear patient response. For instance, if the adolescent did not respond or did not comprehend, and the clinician used “prompt further if required” (question 4), this led to a higher score. The clinicians used all or some of the follow-up questions in the CFI.

*Word matching* was a challenge during the CFI. In some of the T1-core CFI the adolescents talked about several types of problems. In these cases, it seemed challenging for the clinicians to select a problem formulation that could be used and repeated as ‘the problem.’ In one session, the adolescent found it difficult to find a word for the problem and the clinician chose to move on to the next question. In several of the sessions, the clinicians explained that they preferred using the words “problem”, “the cause”, “what you’re describing here” or “the challenge” whenever the CFI question stem included the term “[PROBLEM]”.

### 3.3 DIP and semi-structured interviews with adolescents

All six adolescents answered the DIP questionnaire and the average score on all items was positive. The DIP results are presented in **Table 3**.

**Table 3:** Adolescents’ scores on the debriefing instrument for patients Debriefing Instrument for Patients (DIP) (23), N = 6.

Factor	Item	Mean	Range	Average mean of factor items
Clinical Utility	Helped me explain my main concerns	0.66	-1-1	0.672
	Helped me communicate important aspects of my background/childhood, such as religious faith and/or culture	1.0	1	
	Helped me understand how my background/childhood and current situation affect my problem right now	1.0	0-1	
	Helped me explain what kind of help I would like	0.5	-1 -1	
	Encouraged me to share important information that I might not have mentioned otherwise	0.2	-1 -1	
Feasibility	Questions were easy to understand	1.0	1	1.165
	Took more time to share my perspective than I wanted (reverse scored)	1.33	1-2	
Acceptability	Overall perception of questions	7.5	6-9	

For factor scores of Clinical Utility and Feasibility was scored: -2 = strongly disagree, -1 = disagree, +1 = agree, and +2 = strongly agree. The Acceptability factor score was from lowest 0 to highest 10.

The average mean of the Clinical Utility factor was 0.672, with individual items ranging between the lowest (0.2), “Encouraged me to share important information that I might not have mentioned otherwise”, and the highest (1.0) “Helped me communicate important aspects of my background/childhood, such as religious faith and/or culture” and “Helped me understand how my background/childhood and current situation affect my problem right now”.

The average mean of the Feasibility factor was 1.165. All adolescents answered “Agree” that the questions in the core CFI were easy to understand. The two cases of “Strongly Agree” were for the item “Did not take so much time”.

The six adolescents also answered a question about overall perception of the core CFI. They rated it on a scale from 1 “too personal” to 10 “Good, helped me tell my story”. Overall perception of the core CFI questions had an average mean of 7.5 out of 10.

#### 3.3.1 Regarding the two open DIP questions

Question 1; “Were there any questions that bothered you or made you uncomfortable?”. Four adolescents answered “no” that some of the core CFI questions had bothered them or made them feel uncomfortable, while two responded “yes”. There were follow-up questions “Which ones” and “Why” if they answered “yes”. One adolescent referred to the question about self-coping; “11 and to questions about background” because they were “personal”. Another adolescent wrote “questions about friends and such”, “Because I think it’s painful and embarrassing to say that I don’t have any good friends”.

Question 2; “What other questions might have helped you explain your problem?”. The answers were; “No”; “Can`t think of anything”; “No others questions come to mind right now”; “I don`t know what kind of questions, but that the questions should be less open and more specific”; “For most people, I think the questions help a lot to make them think in new ways and come up with new solutions” and one adolescent had not written anything.

#### 3.3.2 Semi-structured interviews with adolescents

This section is presented in **Table 4** with a summary of the semi-structured interviews with adolescents conducted in two phases. The first interview (T2) took place shortly after the T1- core CFI, and the second (T3) was conducted near the end of the inpatient stay, just before discharge. The analysis resulted in four main themes: treatment experiences, communication with clinicians, experiences of adolescents,’ own involvement, and experiences with the core CFI.

**Table 4:** Overview of semi-structured interviews with adolescents.

Interview time and main themes	Concern	Quotes
T2 Treatment experiences	-mixed feelings about their stay at the unit, balancing homesickness with positive aspects -to address difficult issues was challenging	“Sometimes I want to be here other times, I don’t.” “It gets intense with what I’m struggling with, and it’s touched upon.”
T3 Treatment experiences	-positive experiences, marking the beginning of a process -dissatisfaction with the support, expecting more or that it was different from what they expected	“It has been helpful, and I’ve gotten a small start.” “Hoping to be able to manage symptoms better.” "Now that I have been here, I am actually quite dissatisfied in a way. I had different expectations."
T2 Communication with clinicians	-communication was generally good, describing the clinicians as personable, genuine, and attentive -misunderstandings arose due to inconsistent information-sharing between staff shifts	“I’m happy to get help and that people listen, so I find it easier to open up.” “When I came back, no one had heard about it.”
T3 Communication with clinicians	-communication improved over time -preferring that clinicians took time to listen and were attentive to both medical and personal issues -appreciating being treated as “normal” -misunderstandings occurred suggesting sharing answers and information more clearly and direct	“It has been easy to talk to them, they listen well.” “I feel better with people who talk to me normally.” "There are sometimes small misunderstandings, but for the most part, there aren’t any. Messages could be conveyed more promptly."
T2 Experiences of adolescents’ own involvement	-having a voice in the treatment process -the importance of feeling respected and valued for their opinions in an empathic manner -appreciating the clinicians’ efforts to explore their preferred activities (e.g., weekly activities)	“It’s important that I get to express my opinions.” “If, for example, I have wishes to do activities, they are noted down and so on.”
T3 Experiences of adolescents’ own involvement	-being involved in decisions and allowed to proceed in their own way -in one case, feeling ignored regarding a request to return home	“If I have an opinion it’s heard and addressed right away.” “I’ve said I don’t want to be here so many times to the people around me that I no longer want to say it.”
T2 Experiences with the core CFI	-positive experiences: helping them to share feelings from everyday life, prompting deeper reflections -appreciating the structure, feeling heard and seen by the clinician -the directness made it difficult to answer, suggesting seeing the core CFI in advance in order to be prepared	“I had to think carefully. I knew the answer, but it was hard to formulate.” “It’s better to get one question at a time, so you don`t worry about more questions, like difficult things from childhood.” “I don’t really like answering questions that are straightforward like that, my brain gets dizzy.” "It would have been very helpful to get some kind of overview of the questions in advance."
T3 Experiences with the core CFI	-recalling details from the CFI was difficult, being unsure whether the CFI information had been used or not -helping the clinician understand the difficulties -having an empowering effect, and feeling listened to and acknowledged	“I don’t really remember what I said.” “I managed to say more about what is difficult.” “I think it’s very important that kids are listened to. I hope the interview can help in that way.”

### 3.4 DIC and semi-structured interviews with clinicians

The clinicians answered the DIC questionnaire, and the average mean of factor items were positively rated. The DIC results are presented in **Table 5**.

**Table 5:** Clinician ratings on the debriefing instrument for clinicians Debriefing Instrument for Clinicians (DIC) (23), N = 6.

Factor	Item	Mean	Range	Average mean of factor items
Clinical Utility	Helped me understand the patient’s cultural background	0.6	-1 - 2	1.05
	Clarified the patient’s ideas about the causes of the problem	1.0	-1 - 2	
	Clarified my understanding of the patient’s symptoms and problems	1.16	-1 - 2	
	Gave me confidence in the diagnosis	0.6	-1 - 1	
	Facilitated treatment planning and implementation	1.5	1-2	
	Helped me identify issues that could interfere with treatment adherence (compliance)	1.2	-1 - 2	
	Helped me identify additional aspects or dimensions of the patient’s clinical problem	1.33	1-2	
	Helped me assess the severity of the patient’s problems	0.83	-1 - 2	
	Facilitated my rapport with the patient	1.16	1-2	
	The questions were useful overall	1.16	-1 - 2	
Feasibility	The questions were easy to administer	1.6	1-2	1.48
	The questions were easily understood by the patient	1.5	1-2	
	Contributed positively to the flow of my clinical interview	1.33	1-2	
	Could be integrated easily into my routine clinical practice	1.5	1-2	
Acceptability	Helped make the patient feel more at ease during the interview	0.8	-1 -2	1.34
	Can be incorporated by mental health clinicians into routine clinical interviews	1.6	1-2	
	Facilitated a good assessment of cultural factors relevant to clinical care	1.16	-1 - 2	
	I would recommend for use by other mental health clinicians	1.83	1-2	
The instructions for the interview	Instructions were clear and easy to understand	1.5	1-2	1.5
	Instructions prepared me well to administer the CFI questions	1.5	1-2	

For factor scores of Clinical Utility, Feasibility and Acceptability was scored: -2 = strongly disagree, -1 = disagree, +1 = agree, and +2 = strongly agree.

The average mean of the Clinical Utility factor was 1.05, with individual items ranging between the lowest (0.6) “Gave me confidence in the diagnosis” and the highest (1.5) “Facilitated treatment planning and implementation”. For the item “The questions were useful overall”, the average mean was 1.16.

The average mean of the Feasibility factor was higher, at 1.48. To the open-ended question “What would the challenges be to incorporate the CFI questions into your routine clinical practice?” three clinicians wrote that time must be set aside.

The average mean of the Acceptability factor was 1.34. The question “I would recommend CFI for use by other mental health clinicians” had the highest mean of all individual items at 1.83. Here, all responses were positive (strongly agree or agree). On the item “I would recommend for use by other mental health clinicians”, five clinicians answered “Strongly agree” while one answered “Agree”. Two additional questions about the instructions for the interview had a positive mean score of 1.5.

To the open-ended questions “Which of the CFI questions were the most useful?”, three clinicians wrote that question 1 “What brings you here today?” was useful. Two wrote that question 14 “What kinds of help do you think would be most useful to you at this time?” was useful.

#### 3.4.1 Semi-structured interviews with clinicians

This section, **Table 6**, presents a summary of important themes from the semi-structured interviews with clinicians related to feasibility, acceptability, and clinical utility.

**Table 6:** Overview of important themes from the semi-structured interviews with clinicians related to feasibility, acceptability, and clinical utility.

Efficacy	Main themes	Concern	Quotes
Feasibility	The importance of CFI training	-the CFI training with adjustments of the core CFI provided safety and knowledge	“I am confident about the question order. If I didn’t know it so well, I might not have waited for answers.”
	Complexity in problem formulation	-formulating the problem could be difficult due to the adolescents’ complicated descriptions that included various complex problem areas	“It got so long and clumsy, I couldn’t use it.”
	Time constraints in using the core CFI	-finding a balance between asking supplementary questions and maintaining adolescents` engagement was challenging -sufficient and convenient time for the adolescent must be prioritized	“I asked the supplementary questions I wanted, but it is a dilemma how much extra to ask.” "The timing was chosen so she wouldn’t be tired, which was wise.”
	Understanding and supporting adolescents needs	-the adolescents shared a great deal of personal information -change in the clinicians’ ability to better understand and therapeutically bond with the adolescents -ensuring the adolescents` wellbeing after the interview and follow up by the team	“She expressed what helped her and what made things worse, like her experience with parents and therapists during meals.” “She became very open and looked at me. I am wonder if she felt the need to meet me again.” “He said a lot without knowing me well. It is important the team reads this to understand how to relate to him.”
Acceptability	Different expectations and evaluations of the interview at different time points	-need for reflection on the interview process and content throughout the treatment process	“I got less out of it initially, but the transcription showed good questions, and that the adolescent managed to say important and difficult things about school.”
	The importance of knowing the adolescent	-familiarity with the adolescent prior to the interview could make it easier to extract information from the interaction	“At first the girl`s answers seemed unimportant as she tried not to cry. Knowing her better might have made it easier.”
Clinical Utility	The core CFI gave new information	-positive experiences of using the core CFI, despite the questions being somewhat different from those they normally used	“The interview was surprisingly positive. There was perhaps a bit of concern that there were many questions, standardized questions, somewhat long questions, and concerns about whether the boy would get tired of answering. But it did not happen.”
	The core CFI brought out nuanced diagnostic insights and the adolescents` perspectives	-the T1- core CFI did not change any diagnoses in these patients but provided insights that nuanced the existing ones -information from the adolescent’s perspectives and their narratives created a richer picture for the clinicians	“The diagnosis of an eating disorder is clear, but it helped me understand its multifactorial background better.” “Hearing their self-insights was a aha moment and truly inspiring.”
	The core CFI was respectful and the structure helpful	-the core CFI encouraged a respectful approach towards the adolescents -the question structure from 1 to 16 was helpful for maintaining clear boundaries during the interview session	“The questions and framework are respectful, focusing on the the adolescents’ experiences and their descriptions.” “Knowing what`s coming feel safe. The structure flows well with thoughtful questions.”

### 3.5 Group meetings with clinicians and unit manager

This section, **Table 7**, presents a summary of four group meetings with clinicians and the unit manager, providing an introduction to the implementation process for the pilot study.

**Table 7:** Overview of group meetings with clinicians and unit manager.

Interview and aim	Concern	Quotes
1. At the beginning of the research process To get to know the clinicians, the unit manager, and the clinical context	**- the term “culture” was linked to the adolescents’ context, including family and support systems, but also to the world of the clinic itself** **- clinicians emphasized service user participation and a holistic approach**	“The inpatient unit feels like a small world, a greenhouse with safety and predictability.” “We now focus even more on user participation, but respect for the adolescents has always been there.”
2. Halfway through the research process To evaluate the process midway	**-the adapted core CFI worked well; no further adjustments needed** **- CFI in treatment planning such as timing for sharing CFI information** **-practical barriers to administration of the CFI such as documenting it in medical records**	“I’ve never had such a good conversation with that adolescent before. It was a positive experience.” "I often refer to things from the CFI in meetings, but I am unsure if the team uses it." "It took some time to extract and document, but it was meaningful work.”
3. At the end of the research process To evaluate the efficacy of the 18 patient interviews and provide feedback on the process	**- the main contribution of the core CFI was its ability to strengthen adolescents’ voices** **-more aware of service user participation** **-regarding the administration of the core CFI, clinicians highlighted the importance of flexibility**	“The questions prompt reflection, and bring up things we wouldn’t otherwise ask.” “We focused so much on weight gain and missed other important things, which she expressed in the CFI.” “Prioritizing interviews in the morning, not after school when they might be tired, was important.”
4. After completion of the pilot research project, to complete the research project	**-for the future use of the core CFI, the unit manager emphasized the importance of implementing the core CFI for all adolescents**	“The CFI brings forth adolescents’ opinions, experiences, their thoughts, and voices.”

## 4 Discussion

The aim of the present study was to test the efficacy of the core CFI in terms of feasibility, acceptability, and clinical utility for both adolescents and clinicians in a specialized mental healthcare inpatient unit in Norway. This is in line with existing calls for CFI improvement (44, 45), and the need for studies on adaptations to adolescents to improve the cultural responsiveness of mental health services (56). Existing research on the CFI is limited regarding majority populations (44) and adolescents (43). To our knowledge, this is the first study to test the efficacy of the core CFI for adolescents in mental healthcare. The format from the field trials (23) was modified to assess the core CFI and its usage throughout the treatment process for adolescents, integrating an organizational level with cultural analysis and feedback. This was described as the CFI process in this study. The discussion is organized around central points related to the findings of this pilot efficacy study.

### 4.1 CFI training and Fidelity

Research suggests that clinicians can be trained effectively in the use of the core CFI (40, 46). This concurs with the systematic review by Gondek et al. (15), which found that effective PCC requires clinicians to have confidence and relevant knowledge and that this can be achieved through training. To ensure fidelity to the core CFI, interviewers need to adhere to the core CFI script, pose personalized follow-up questions for clarification, and accurately reflect participants’ experiences (35, 71). In this study, the clinicians delivered the core CFI with a high fidelity score. Essential for this degree of fidelity was the multi-level CFI training process that included cultural-competence training. This finding is in line with that of the study conducted in the Netherlands (49), which highlighted that effective implementation of the CFI was closely linked to the level of training and cultural competence of clinicians. The study found that clinicians with better cultural training and experience were more likely to use the CFI effectively, reinforcing the importance of thorough training in achieving high fidelity in administering the core CFI (49).

In the present study, the Cultural Formulation Interview – Fidelity Instrument (CFI-FI) for clinicians conducting the CFI showed that patient-centeredness received a high score, indicating that the clinicians maintained a respectful and non-confrontational attitude. This aligns with the introductory principle of the core CFI that there are no right or wrong answers (16). Clinicians adhered well to the structure of the core CFI, but challenges were noted in word matching regarding problem formulation. Clinicians sometimes found it difficult to select a problem formulation for further exploration as the problem areas were diverse. In the semi-structured interviews with the clinicians, this was highlighted as a particular problem. The difficulty arose when adolescents used extensive descriptions, resulting in long “clumsy” formulations. This finding addresses the need for integrating this problem area in CFI training in the future.

The CFI training in the present study included time for discussion of each question and possible adjustments for the patient group. Imbedding in the training a cultural competence dimension, including the clinician’s exploration of her/his own cultural information, proved essential for bringing the concepts of culture and cultural information into a working framework. The findings indicated that the level of CFI training was appropriate and adequate. The findings also demonstrated that the interviews and meetings with clinicians, addressing self-reflection and communication, were of importance for how the information from the core CFI was applied in the adolescents’ treatment. These results correspond with the study by Aggarwal et al. (46), where clinicians felt that training helped them to learn how to ask the CFI questions and resolve their doubts about the interview. The findings support DeSilva et al. (42) and Wallin et al. (28), who find it crucial to adjust the core CFI to make the questions more comprehensible in relation to ongoing clinical activity.

While primarily an assessment tool, the CFI has also been described as a communication intervention with potential therapeutic effects (40, 71). The CFI elicits clinically relevant information that fosters deeper understanding and facilitates meaningful dialogue between clinicians and patients (48). The CFI is described as an intervention rooted in cultural competence training (71). These therapeutic effects may occur because the CFI provides a framework for structuring conversations about culture, context, and identity, enhancing the clinician’s ability to engage patients in person-centered care. The key themes related to the CFI as an intervention, as outlined in the *DSM-5 Handbook on the cultural formulation interview* (28), include: 1) cultural identity of the individual, 2) cultural explanations of the individual’s illness, 3) cultural factors related to psychosocial environment and levels of functioning, and 4) cultural elements of the relationship between the individual and clinician. To date, there is no further research regarding the precise nature of the CFI as an intervention. Through this clinical CFI study, as well as the results in other clinical contexts using the same approach, an examination of the CFI as a type of brief Intervention is now underway.

### 4.2 Efficacy assessments by adolescents

The DIP questionnaire showed that the six adolescents gave positive to very positive ratings for clinical utility, feasibility, and acceptability.

The clinical utility of the core CFI, assessed through DIP responses and qualitative interviews, demonstrated positive experiences among the adolescents. Some of them mentioned that the questions had prompted deeper reflection. This is in line with the finding of Muralidharan and colleagues (35) that most patients felt that they had to think carefully to give a proper answer. Such findings align with existing literature emphasizing adolescents’ desire to have an active role in their treatment process and the importance of their views being respected and valued (10–12). While the majority expressed satisfaction with their involvement in decisions, instances where given information was not shared among clinicians led to misunderstandings and a sense of being unheard. This highlighted a need for ongoing efforts to ensure meaningful service user involvement. The study indicates that the core CFI played a crucial role in providing valuable information, aiding communication about significant aspects of the adolescents’ backgrounds. The positive acceptability of the core CFI in our study is consistent with literature highlighting its value in fostering open communication about sensitive topics (33, 35, 38). However, challenges were noted in recalling CFI information during T3 interviews, raising concerns about the consistency of integration of the CFI information into treatment (44). This underscores the importance of ensuring that adolescents do not feel unheard or overlooked in the treatment process.

The adolescents’ exploration of feasibility, as assessed through the DIP and the qualitative interviews, showed that they had positive views of the core CFI. In the DIP questionnaire, immediately after the T1- core CFI, the adolescents indicated that they found the questions easy to understand. This perception was corroborated by clinicians in response to the statement about the CFI questions, “Were easily understood by the patient” in the DIC questionnaire. The high agreement from DIP, including two adolescents strongly agreeing that the T1- core CFI did not take up much time, highlights the feasibility of incorporating the core CFI into the routines of the inpatient unit. This positive finding corresponds with existing literature emphasizing high patient satisfaction, trust building, and engagement in care through the use of the core CFI (33, 34, 38).

During the T2 interviews, at a later point in the treatment process, the adolescents repeated the finding from DIP that they found the CFI questions to be positive. Several added that the questions were positive despite finding some questions challenging because the questions were different from what they had been asked in other interviews, and required them to think at a deeper level. The CFI set in motion a reflection process for the adolescents. Challenging questions were not easy questions to answer, but were important questions that required deeper thought to answer and stayed with the adolescents in their therapeutic process at the clinic.

The acceptability of the core CFI was generally positive in DIP and the semi-structured, qualitative interviews that followed. Some adolescents expressed discomfort with specific questions due to their personal character. Some challenges were raised by the adolescents. In response to the open-ended question in the DIP questionnaire about whether any questions bothered them or made them feel uncomfortable, two adolescents provided feedback. One adolescent mentioned that question 8, about background, and question 11, about self-coping, felt too personal. Another adolescent pointed out that questions about friends were “painful and embarrassing to say that I don’t have any good friends.” This feedback is crucial for the clinical team to consider, as it touches on vulnerable aspects of the adolescents’ lives. This stresses the importance of awareness of the potentially sensitive nature of certain CFI questions for adolescents, especially for those lacking a network, friends, or family. For some, the directness and unfamiliarity of the questions made it challenging to articulate their feelings. However, allowing more time for the self-reflecting questions gave several participants the ability to answer the questions with important information. As one patient mentioned it might be worth considering potential benefits of providing an overview of the questions in advance to help adolescents be more prepared. However, the core CFI offered a positive framework, providing a structured and secure environment for addressing these sensitive topics. This dual role of the core CFI recognizes its potential sensitivity while highlighting its ability to facilitate narratives on challenging subjects for adolescents. Dixon and colleagues (22) emphasize the importance of including the person’s own culture, background, and goals in treatment planning.

The adolescents reported in the follow-up interviews (T3) at the end of treatment that the core CFI facilitated a nuanced exploration of their backgrounds and difficulties, providing clinicians with valuable insights for the therapeutic process. The insights gained through the CFI by the adolescents set in motion a process of reflection reported on later in the therapeutic process. This finding supports the use of referring to the CFI experience as the CFI process. The desire for meaningful patient involvement observed in our study resonates with recommendations for PCC where the patient’s voice is integral to the treatment approach (14, 15). For both patients and clinicians inclusion of cultural information throughout the diagnostic and treatment processes, and both returning to as well as augmenting cultural information from the core CFI throughout the treatment process, provides additional support for a multi-step CFI process (56).

### 4.3 Efficacy assessments by clinicians

The clinicians’ exploration of **feasibility**, as assessed through the DIC and the semi-structured interviews, provided valuable insights into practical aspects of the core CFI. The specified CFI training was seen as essential for applying the core CFI in a person-centered manner, especially in terms of creating a safe interview space, ensuring that the words and expressions were suitable for this group of adolescents, allowing the adolescents time to find their answers, and maintaining a non-judgmental attitude. Despite overall positive feedback from the clinicians, one challenge was time constraints in using the instrument. This barrier to implementing the core CFI was reported in studies from the DSM-5 international field trials (23) and in India (74). With practice, the core CFI takes approximately 20 minutes to complete (37). In this study, the T1- core CFI ranged from 22 to 59 minutes, with an average duration of 37 minutes. The emotional impact on the adolescents was acknowledged, emphasizing the need to ensure their well-being after the T1- core CFI. The timing of the T1- core CFI was not always optimal for the adolescents, as they could be tired after school and treatment sessions. A suggestion was to conduct the core CFI early in the day when adolescents have greater capacity for it.

Acceptability of the core CFI was generally positive in DIC and the qualitative interviews. The positive responses indicated overall acceptability among clinicians, highlighting its potential as a valuable tool in mental health settings. The clinicians would recommend the core CFI to other mental health professionals working with adolescents.

The clinical utility of the core CFI, assessed through DIC responses and qualitative interviews, demonstrated positive experiences among clinicians. The tool was perceived as providing new and nuanced information to the diagnostic process. The CFI offered insight into the adolescents’ narratives, fostering a deeper understanding of their experiences. The clinicians highlighted the respectful approach of the core CFI when exploring the adolescents’ perspectives. By paying attention to the adolescents’ understanding of their illness, they could build trust and an alliance, both being key elements in treatment (28, 33, 35, 38, 41, 44).

Overall, the clinicians found that the core CFI and the CFI process strengthened the adolescents’ voices in their treatment. The findings highlight the positive impact on communication, service user involvement, and the overall treatment experience.

### 4.4 Challenges identified in the CFI process

Overall, the results showed that the adolescents’ and clinicians’ perceptions of the feasibility, acceptability and clinical utility of the core CFI were very positive. Use of the core CFI with a majority ethnic group appears to support the international evidence that information from the CFI l can contribute to the areas of service user involvement and treatment planning for all patients (35, 38, 53, 74). These assessments of efficacy have been focused on the core CFI experience itself. However, this study did not end with the core CFI experience but encompassed the CFI process, examining how insights and information from the core CFI could be integrated into clinical practice. Though the CFI process worked in different ways during the treatment process, it is important to note the types of challenges identified in numerous clinical team meetings and interviews with clinicians.

Challenges have arisen regarding how to optimize the suitability of the core CFI in various healthcare settings (44). In the present study, the clinicians’ reflections from group meetings and discussions with the unit manager underlined the broader impact of the core CFI on the clinical team. There were a number of challenges involved. One challenge was the time required to document and distill the CFI findings into the medical record. Although clinicians found the process meaningful, it was described as time-consuming and potentially burdensome in the context of a busy clinical environment. For instance, it was seen as essential to determine how the CFI information could be seamlessly integrated into team-based care, especially in situations where other clinicians are involved in diagnostic assessments and treatment decisions. Another challenge was related to the timing of the CFI interviews. Clinicians emphasized the importance of scheduling interviews at times when adolescents were most receptive and alert, such as in the morning rather than after school when they might be tired. This adjustment was seen as critical for ensuring that the interviews were effective and meaningful. Additionally, some clinicians expressed uncertainty about how effectively the CFI information was being utilized by the broader team. While some clinicians frequently referred to CFI insights in team meetings, it was unclear whether this information was consistently integrated into treatment planning by all team members. This highlights the need for improved communication and coordination to ensure that the full potential of the CFI is realized in team-based care. Clinicians also pointed out the importance of flexibility in administering the CFI. For example, they noted that the structure of the interview should allow for adaptation to the adolescent’s individual needs and circumstances, ensuring a person-centered and contextually relevant approach. These results correspond with the study by Wallin and colleagues (28–30), who found that not sharing information to all members in a team could limit the potential of the core CFI, underscoring the need to ensure comprehensive information sharing for all patients. To address this particular challenge, extra team meetings were organized relating to documentation processes and establishing standardized procedures for integrating CFI findings into treatment plans. A clinical implication of this finding is to pay attention to barriers and resources related to communication levels in team-based treatment.

In the semi-structured interviews with the adolescents, two types of challenges were identified. The first pertained to dissatisfaction with the support they received, as it did not meet their expectations. The second type of challenge was related to misunderstandings in communication, caused by inconsistent information-sharing between staff shifts and unclear messaging. In this context, one adolescent suggested that answers and information should be shared more clearly and directly.

These findings align with PCC research, which highlights communication skills as a central component (4, 5). This includes active listening, compassionate and empowering care, respect for patient autonomy, and sensitivity to patients’ needs. However, in Norway, reports indicate that legislation and rights for adolescents are not always fully realized (62–65). This underscores the growing demand for improved communication skills within adolescent mental healthcare (14, 15).

### 4.5 Study contributions

The present efficacy study of the core CFI is a key contribution to PCC research, corresponding to a PCC approach where adolescents’ desire for active engagement in their treatment processes is taken seriously (14). Furthermore, the present study is a contribution to CFI research on adolescents in mental healthcare, which has been limited, compared to the adult population (51–55). Aggarwal et al. (26) have highlighted the positive impact of the core CFI on communication and therapeutic rapport, yet the practical utilization of this information remains a nuanced aspect that requires careful consideration. Our study contributes to this discourse by shedding light on the balance between obtaining crucial information and ensuring that adolescents feel heard and acknowledged throughout their treatment. Finally, the present study is a contribution to research on specific strategies and adaptations for adolescents to improve the cultural responsiveness of mental health services (56).

### 4.6 Clinical implications

A clinical implication of this study is related to introducing the core CFI to a new facility with training, clinician fidelity and ongoing supervision (32, 40, 42, 49). Supervision refers to continuous organizational-level guidance for clinicians to help them understand and effectively communicate the impact of culture on mental healthcare, and to apply CFI information to patient care (32). Additionally, ensuring mandatory participation in training for CFI implementation in a clinical context is essential for maintaining clinician engagement and commitment to culturally responsive care (49). This clinical study can be regarded as an organizational study as well, due to the cultural analysis of the clinic’s work culture and that of its larger organization, close collaboration with clinicians and unit manager, and feedback to the unit. This provided insight into the organizational dynamics and cultural factors influencing the CFI process. The close collaboration with the unit was beneficial in that the first author had for many years, providing an insider perspective, while the other two authors brought outsider perspectives. However, this insider-outsider dynamic may have introduced bias or influenced the interpretation of results. When incorporating the core CFI, it is important to focus on individual patients’ experiences and allow them to tell their own story (44). Clinicians have to remain culturally sensitive throughout treatment (43) and training in what is meant by culture in the CFI is therefore essential (42). The present study highlights the importance of considering the potentially sensitive nature of certain CFI questions, emphasizing the need for a thoughtful and adaptable approach. The clinicians highlighted that the core CFI should be administrated in a flexible way in relation to the schedule of the treatment, not only in the initial assessment as recommended (67). Thus, this study’s results are aligned with the qualitative studies by Wallin et al. (28) and Skammeritz et al. (34), suggesting that clinicians need flexibility in the time spent on administering the core CFI. Clinicians can find value in CFI information at any stage of the patient’s treatment, provided that the patient is in a clinically stable condition and healthcare staff acknowledge the importance of the CFI and actively engage in its effective integration (44, 50).

### 4.7 Future research

To enhance the evidence base, future research should consider conducting a RCT to compare outcomes between adolescents who receive the core CFI and those who do not. Additionally, extending the testing of the core CFI to diverse clinical contexts, such as outpatient clinics and primary healthcare for adolescents, would provide a more comprehensive understanding of its effectiveness in varied settings. These research directions can provide valuable insights into the broader applicability and impact of the CFI process, further informing evidence-based practices in adolescent mental healthcare.

### 4.8 Strengths and limitations

The study sample was limited in order to apply the extended CFI protocol, providing efficacy testing at several points in time during the adolescents’ treatment. This approach, described as the CFI process in this study, provided valuable insights related to efficacy as operationalized through feasibility, acceptability, and clinical utility assessments. While the small sample size of six consecutive patients allowed for detailed and in-depth exploration of the CFI process, it also limits the generalizability of the findings. Additionally, the range of perspectives captured may have been too narrow to comprehensively examine complex constructs such as cultural identity, treatment engagement, and meaning-making. The methodological format of the study followed the CFI process, including patient interviews at three time points, which allowed for a longitudinal perspective on the adolescents’ treatment experiences. The credibility of the findings was further strengthened by an in-depth analysis of the material, conducted collaboratively by the authors at every stage of the analytical process. This collaboration ensured a balanced and comprehensive analysis, contributing to the robustness of the study’s conclusions. The study’s emphasis on efficacy underscores its exploratory nature, highlighting the need for future research with larger and more diverse samples to validate and expand upon these findings.

As a pilot study, using this methodology the small and homogeneous sample is justified. However, there are limitations. It is important to acknowledge that positive findings, such as high acceptability ratings, may have been influenced by selection bias, as consecutive participants willing to engage in the study might have been more positively predisposed toward the CFI. Additionally, clinician enthusiasm during training and implementation may have contributed to favorable evaluations. The phased approach of testing the core CFI with three adolescents aged 14–15 before broader implementation, ensured the core CFI suitability and allowed for necessary adaptations in collaboration with clinicians. The decision to not include **Supplementary Module 9** for “School-age children and adolescents” (68), was grounded on the testing of the core CFI with adolescents at the clinic and discussions with clinicians finding the that core CFI with adaptations was sufficient. This iterative process enhanced the reliability of the method (44).

This qualitative study did not include a control group, which is a clear limitation. To the degree possible, similar patient files were reviewed by the clinical staff and notations were made as to the difference in the kind of information or nuances in information that emerged through the CFI process for treatment planning as well as the degree of adolescent engagement in treatment.

The study demonstrated that clinicians became more confident and comfortable with administering the CFI as they gained familiarity with the questions over time. This progression improved the flow and depth of the interviews, emphasizing the importance of practice and experience in effectively utilizing the CFI (44).

The data collection period coincided with a challenging situation in the inpatient unit. There were ongoing staff restructuring processes, implementation of new treatment methods, changes in staff composition, changes in leadership, and critical specialist shortages. Additionally, the clinic faced uncertainties regarding its future, including the potential for closure, which created a sense of instability among staff. These institutional challenges were further compounded by the impact of the Covid-19 pandemic, which involved lockdowns, implementation of infection control measures, and illness among the staff. These factors directly impacted the recruitment process by reducing the overall number of eligible adolescents admitted to the unit, and limiting the availability of clinicians to participate in the study. However, the original study design, which included six consecutive adolescent patients and 2–3 clinicians, was not altered to accommodate these challenges. Instead, adjustments were made to the timing of CFI administration to accommodate both clinicians’ and patients’ schedules, contributing to variability in the clinical timing of the data collection process.

Deductive analysis was used, following the analysis process used in the field trials. While this approach allowed for consistency with the established protocol, it may have limited the discovery of nuances that could have emerged through an inductive analytical process. Adherence to the predefined factors of efficacy might have meant that other aspects of the adolescents’ and clinicians’ experiences were overlooked. Despite these limitations, it is important to note that ongoing interviews with clinicians at different stages of the research process, and interviews with adolescents before completion of treatment provided additional insights and complemented the data obtained from the T1- core CFI.

## 5 Conclusion

To our knowledge, this is the first study to test the efficacy of the core CFI for adolescents in mental healthcare. Previous studies on the CFI with adolescents (59–63) have explored its use in case studies, family engagement, and culturally informed care models but have not assessed specifically its efficacy in terms of feasibility, acceptability, and clinical utility. Overall, the results showed that the adolescents’ and clinicians’ perceptions of efficacy of the core CFI were positive. Use of the core CFI with a cultural majority group of adolescent patients appears to support the international evidence that information from the CFI can contribute to the areas of service user involvement and treatment planning with all patients.

This study highlights a structured systematic process for implementing the core CFI in clinical practice. Beyond simply administering the tool, following a systematic process allowed for clinician adaptation and refinement, contributing to the successful integration of the CFI (44). Testing the core CFI with a small group of adolescents aged 14–15 in the early stages of the study provided valuable insights that informed subsequent adaptations, ensuring that the method was age-appropriate.

This person-centered approach of the core CFI provides valuable knowledge for future adaptations and broader integration into clinical practices. The findings underscore the significance of progressive clinician training and experience in achieving effective use of the core CFI. As clinicians became more familiar with the tool, their ability to engage adolescents and elicit meaningful narratives improved, reinforcing the value of embedding the core CFI within a well-supported and iterative implementation process. These results provide a strong foundation for refining the core CFI to suit diverse contexts. Furthermore, the pilot findings suggest that the systematic use of the core CFI could be tested in broader controlled trials to evaluate its impact on clinical outcomes, including service user involvement, treatment planning, and therapeutic alliance across diverse populations and settings. Wallin and colleagues (31) provide an example of how adding the CFI to routine diagnostic procedures facilitated the identification of psychiatric symptoms, such as depression, particularly among non-native speaking patients. Their study also emphasized the importance of integrating the CFI into diagnostic practices to optimize its clinical utility. Building on such research, future trials could explore the CFI’s broader applicability in diverse clinical contexts to validate its utility further.

## Data Availability

The original contributions presented in the study are included in the article/**Supplementary Material**, further inquiries can be directed to the corresponding author/s.

## References

[1] Morgan S Yoder LH . A concept analysis of person-centered care. J Holist Nurs. (2012) 30:6–15. doi: 10.1177/0898010111412189, PMID: 21772048

[2] Davidson L Tondora J . Person-centred care planning as foundational to clinical practice. World Psychiatry. (2022) 21:1–2. doi: 10.1002/wps.20922, PMID: 35015347 PMC8751555

[3] World Health Organization. Guidance on community mental health services: Promoting person-centred and rights-based approaches (2021). Geneva: World Health Organization. Available online at: https://www.who.int/publications/i/item/9789240025707 (Accessed December 15, 2025).

[4] Santana MJ Manalili K Jolley RJ Zelinsky S Quan H Lu M . How to practice person-centred care: A conceptual framework. Health Expect. (2018) 21:429–40. doi: 10.1111/hex.12640, PMID: 29151269 PMC5867327

[5] Constand MK MacDermid JC Dal Bello-Haas V Law M . Scoping review of patient-centered care approaches in healthcare. BMC Health Serv Res. (2014) 14:1–9. doi: 10.1186/1472-6963-14-271, PMID: 24947822 PMC4079171

[6] Byczkowski TL Kollar LM Britto MT . Family experiences with outpatient care: Do adolescents and parents have the same perceptions? J Adolesc Health. (2010) 47:92–8. doi: 10.1016/j.jadohealth.2009.12.005, PMID: 20547297

[7] Buston K . Adolescents with mental health problems: what do they say about health services? J Adolesc. (2002) 25:231–42. doi: 10.1006/jado.2002.0463, PMID: 12069437

[8] Hart A Saunders A Thomas H . Attuned practice: a service user study of specialist child and adolescent mental health, UK. Epidemiol Psichiatr Soc. (2005) 14:22–31. doi: 10.1017/s1121189x00001895, PMID: 15792291

[9] Svamo NTØ Haug SHK DeMarinis V Hertzberg U . Adolescents’ voices on self-engagement in mental health treatment: a scoping review. Eur Child Adolesc Psychiatry. (2024) 33:4083–95. doi: 10.1007/s00787-024-02425-7, PMID: 38538878 PMC11618195

[10] Coyne I McNamara N Healy M Gower C Sarkar M McNicholas F . Adolescents' and parents' views of Child and Adolescent Mental Health Services (CAMHS) in Ireland. J Psychiatr Ment Health Nurs. (2015) 22:561–9. doi: 10.1111/jpm.12215, PMID: 25977175

[11] Ronzoni P Dogra N . Children, adolescents and their carers’ expectations of child and adolescent mental health services (CAMHS). Int J Soc Psychiatry. (2012) 58:328–36. doi: 10.1177/0020764010397093, PMID: 21242172

[12] Midgley N Holmes J Parkinson S Stapley E Eatough V Target M . Just like talking to someone about like shit in your life and stuff, and they help you”: Hopes and expectations for therapy among depressed adolescents. Psychother Res. (2016) 26:11–21. doi: 10.1080/10503307.2014.973922, PMID: 25372575

[13] Stafford V Hutchby I Karim K O’Reilly M . Why are you here?” Seeking children’s accounts of their presentation to Child and Adolescent Mental Health Service (CAMHS). Clin Child Psychol Psychiatry. (2016) 21:3–18. doi: 10.1177/1359104514543957, PMID: 25062687

[14] Viksveen P Bjønness SE Cardenas NE Game JR Berg SH Salamonsen A . User involvement in adolescents’ mental healthcare: a systematic review. Eur Child Adolesc Psychiatry. (2022) 31:1765–88. doi: 10.1007/s00787-021-01818-2, PMID: 34089383 PMC9666298

[15] Gondek D Edbrooke-Childs J Velikonja T Chapman L Saunders F Hayes D . Facilitators and barriers to person-centred care in child and young people mental health services: A systematic review. Clin Psychol Psychother. (2017) 24:870–86. doi: 10.1002/cpp.2052, PMID: 27910173

[16] Lewis-Fernández R Aggarwal NK Kirmayer LJ . Introduction. In: Lewis-Fernández R Aggarwal NK Hinton L Hinton DE Kirmayer LJ , editors. DSM-5® Handbook on the Cultural Formulation Interview. American Psychiatric Publishing, Washington DC (2016). p. xxvii–xiv.

[17] American Psychiatric Association . *Diagnostic and Statistical Manual of Mental Disorders: DSM-5.* 5 ed. Washington, D.C: American Psychiatric Association (2013).

[18] American Psychiatric Association . *Diagnostic and Statistical Manual of Mental Disorders: DSM-5-TR* Fifth Edition. Washington, D.C: American Psychiatric Association (2022).

[19] American Psychiatric Association . Diagnostic and statistical manual of mental disorders: DSM-IV. Washington, D.C: American psychiatric association (1994).

[20] Lewis-Fernández R Aggarwal NK Kirmayer LJ . Cultural Formulation Before DSM-5. In: R. Lewis-Fernández NKA Hinton L Hinton DE Kirmayer LJ , editors. DSM-5® Handbook on the Cultural Formulation Interview. American Psychiatric Publishing, Washington DC (2016). p. 1–26.

[21] Aggarwal NK Jiménez-Solomon O Lam PC Hinton L Lewis-Fernández R . The core and informant Cultural Formulation Interviews in DSM-5. In: Lewis-Fernández R Aggarwal NK Hinton L Hinton DE Kirmayer LJ , editors. DSM-5® Handbook on the Cultural Formulation Interview. American Psychiatric Publishing, Washington DC (2016). p. 27–44.

[22] Dixon LB Holoshitz Y Nossel I . Treatment engagement of individuals experiencing mental illness: review and update. World Psychiatry. (2016) 15:13–20. doi: 10.1002/wps.20306, PMID: 26833597 PMC4780300

[23] Lewis-Fernandez R Aggarwal NK Lam PC Galfalvy H Weiss MG Kirmayer LJ . Feasibility, acceptability and clinical utility of the Cultural Formulation Interview: mixed-methods results from the DSM-5 international field trial. Br J Psychiatry. (2017) 210:290–7. doi: 10.1192/bjp.bp.116.193862, PMID: 28104738

[24] Rohlof H van Dijk RC Groen SP Aggarwal NK Lewis-Fernández R . The Cultural Formulation Interview: Results from the international field trial in The Netherlands. World Cultural Psychiatry Res Rev. (2017) 12:26–37.

[25] Díaz E Añez LM Silva M Paris M Davidson L . Using the Cultural Formulation Interview to build culturally sensitive services. Psychiatr Serv. (2017) 68:112–4. doi: 10.1176/appi.ps.201600440, PMID: 27799018

[26] Aggarwal NK DeSilva R Nicasio AV Boiler M Lewis-Fernández R . Does the Cultural Formulation Interview for the fifth revision of the diagnostic and statistical manual of mental disorders (DSM-5) affect medical communication? A qualitative exploratory study from the New York site. Ethn Health. (2015) 20:1–28. doi: 10.1080/13557858.2013.857762, PMID: 25372242 PMC4221811

[27] Hinton L Aggarwal N Iosif A-M Weiss M Paralikar V Deshpande S . Perspectives of family members participating in cultural assessment of psychiatric disorders: Findings from the DSM-5 International Field Trial. Int Rev Psychiatry. (2015) 27:3–10. doi: 10.3109/09540261.2014.995072, PMID: 25738941 PMC4455874

[28] Wallin MI Dahlin M Nevonen L Bäärnhielm S . Patients’ and clinicians’ experiences of the DSM-5 Cultural Formulation Interview: A mixed method study in a Swedish outpatient setting. Transcult Psychiatry. (2020) 57:542–55. doi: 10.1177/1363461520938917, PMID: 32646300 PMC7488836

[29] Wallin MI DeMarinis V Nevonen L Bäärnhielm S . A qualitative analysis of the documentation of DSM-5 Cultural Formulation Interviews with non-native speaking patients in a Swedish mental health care setting. Front Psychiatry. (2024) 15:1298920. doi: 10.3389/fpsyt.2024.1298920, PMID: 38455521 PMC10918747

[30] Wallin MI DeMarinis V Nevonen L Bäärnhielm S . What information did the DSM-5 Cultural Formulation Interviews provide when used with Swedish-speaking patients in a psychiatric setting in Stockholm? Front Psychiatry. (2024) 15:1377006. doi: 10.3389/fpsyt.2024.1377006, PMID: 38840947 PMC11151123

[31] Wallin MI Galanti MR Nevonen L Lewis-Fernández R Bäärnhielm S . Impact on routine psychiatric diagnostic practice from implementing the DSM-5 Cultural Formulation Interview: A pragmatic RCT in Sweden. BMC Psychiatry. (2021) 22:149. doi: 10.1186/s12888-022-03791-9, PMID: 35216555 PMC8876131

[32] Lindberg LG Carlsson J Kristiansen M Skammeritz S Johansen KS . The Cultural Formulation Interview—Generating distance or alliance? A qualitative study of practice changes in Danish mental healthcare. Transcult Psychiatry. (2022) 59:740–55. doi: 10.1177/13634615211065617, PMID: 35331059

[33] Lindberg LG Johansen KS Kristiansen M Skammeritz S Carlsson J . Negotiating engagement, worthiness of care and cultural identities through intersubjective recognition: migrant patient perspectives on the cultural formulation interview in danish mental healthcare. Culture Medicine Psychiatry. (2021) 45:629–54. doi: 10.1007/s11013-020-09694-2, PMID: 33170411

[34] Skammeritz S Lindberg LG Mortensen EL Norredam M Carlsson J . Using the Cultural Formulation Interview in Denmark: Acceptability and clinical utility for medical doctors and migrant patients. Transcult Psychiatry. (2020) 57:556–66. doi: 10.1177/1363461520935673, PMID: 32838657

[35] Muralidharan A Schaffner RM Hack S Jahn DR Peeples AD Lucksted A . I got to voice what’s in my heart”: Participation in the Cultural Formulation Interview—perspectives of consumers with psychotic disorders. Psychosoc Rehabil Ment Health. (2017) 4:35–43. doi: 10.1007/s40737-017-0076-y

[36] Gearin P Bojdani E Zhang T Ly D Li KJ . Advancing diagnostic accuracy and treatment with the cultural formulation interview: assessment of psychosis in a Jamaican-american woman. Am J Psychiatry Residents' J. (2020) 15:12–4. doi: 10.1176/appi.ajp-rj.2020.150305

[37] Jarvis GE Kirmayer LJ Gómez-Carrillo A Aggarwal NK Lewis-Fernández R . Update on the cultural formulation interview. Focus (Madison). (2020) 18:40–6. doi: 10.1176/appi.focus.20190037, PMID: 32047396 PMC7011218

[38] Ramirez Stege AM Yarris KE . Culture in la clínica: evaluating the utility of the Cultural Formulation Interview (CFI) in a Mexican outpatient setting. Transcult Psychiatry. (2017) 54:466–87. doi: 10.1177/1363461517716051, PMID: 28691591

[39] Aggarwal NK Cedeno K Lewis-Fernandez R . Patient and clinician communication practices during the DSM-5 cultural formulation interview field trial. Anthropol Med. (2020) 27:192–211. doi: 10.1080/13648470.2019.1641014, PMID: 31550913 PMC7093248

[40] Aggarwal NK Jarvis GE Gómez-Carrillo A Kirmayer LJ Lewis-Fernández R . The Cultural Formulation Interview since DSM-5: Prospects for training, research, and clinical practice. Transcult Psychiatry. (2020) 57:496–514. doi: 10.1177/1363461520940481, PMID: 32838655

[41] Paralikar VP Deshmukh A Weiss MG . Qualitative analysis of Cultural Formulation Interview: Findings and implications for revising the Outline for Cultural Formulation. Transcult Psychiatry. (2020) 57:525–41. doi: 10.1177/1363461518822407, PMID: 30636531

[42] DeSilva R Aggarwal NK Lewis-Fernández R . The DSM-5 Cultural Formulation Interview: bridging barriers toward a clinically integrated cultural assessment in psychiatry. Psychiatr Ann. (2018) 48:154–9. doi: 10.3928/00485713-20180214-01

[43] Jones-Lavallée A Bernard G Taing J Leanza Y . The state of current knowledge on the cultural formulation interview: A scoping review. J Psychopathol Behav. (2022) 45:265–76. doi: 10.1007/s10862-022-10009-5

[44] Lewis-Fernández R Aggarwal NK Kirmayer LJ . The Cultural Formulation Interview: Progress to date and future directions. Transcultural Psychiatry. (2020) 57:487–96. doi: 10.1177/1363461520938273, PMID: 32838656

[45] Aggarwal NK Nicasio AV DeSilva R Boiler M Lewis-Fernández R . Barriers to implementing the DSM-5 cultural formulation interview: a qualitative study. Culture Medicine Psychiatry. (2013) 37:505–33. doi: 10.1007/s11013-013-9325-z, PMID: 23836098 PMC4299818

[46] Aggarwal NK Lam P Castillo EG Weiss MG Diaz E Alarcón RD . How do clinicians prefer cultural competence training? Findings from the DSM-5 cultural formulation interview field trial. Acad Psychiatry. (2016) 40:584–91. doi: 10.1007/s40596-015-0429-3, PMID: 26449983 PMC4826320

[47] Mills S Wolitzky-Taylor K Xiao AQ Bourque MC Rojas SMP Bhattacharya D . Training on the DSM-5 cultural formulation interview improves cultural competence in general psychiatry residents: a multi-site study. Acad Psychiatry. (2016) 40:829–34. doi: 10.1007/s40596-016-0551-x, PMID: 27093964

[48] Mills S Xiao AQ Wolitzky-Taylor K Lim R Lu FG . Training on the DSM-5 Cultural Formulation Interview improves cultural competence in general psychiatry residents: a pilot study. Transcult Psychiatry. (2017) 54:179–91. doi: 10.1177/1363461517700812, PMID: 28358239

[49] Silvius L Antezana KV Ghane S . Symptom vs context: Lessons learned from a large-scale implementation of the Cultural Formulation Interview. Front Psychiatry. (2024) 15:1410865. doi: 10.3389/fpsyt.2024.1410865, PMID: 39296860 PMC11408998

[50] Aggarwal NK Chen D Lewis-Fernández R . Eliciting social stressors, supports, and determinants of health through the DSM-5 cultural formulation interview. Front Psychiatry. (2023) 14:1148170. doi: 10.3389/fpsyt.2023.1148170, PMID: 37056400 PMC10086136

[51] La Roche MJ Bloom JB . Examining the effectiveness of the Cultural Formulation Interview with young children: A clinical illustration. Transcult Psychiatry. (2018) 57:515–24. doi: 10.1177/1363461518780605, PMID: 29956584

[52] Rousseau C Johnson-Lafleur J Papazian-Zohrabian G Measham T . Interdisciplinary case discussions as a training modality to teach cultural formulation in child mental health. Transcult Psychiatry. (2018) 57:581–93. doi: 10.1177/1363461518794033, PMID: 30131020

[53] Sanchez AL Jent J Aggarwal NK Chavira D Coxe S Garcia D . Person-centered cultural assessment can improve child mental health service engagement and outcomes. J Clin Child Adolesc Psychol. (2022) 51:1–22. doi: 10.1080/15374416.2021.1981340, PMID: 34905434

[54] Arshad SH Chua J Wayne S-A Bryant JL Al-Mateen CS . Tools to craft a cultural formulation. Child Adolesc PsychiatricClinics North America. (2022) 31:583–601. doi: 10.1016/j.chc.2022.05.001, PMID: 36182212

[55] Shem-Tov RG Zubery E Hecht NL Latzer Y . A full stomach”: culturally sensitive diagnosis of eating disorders among Ethiopian adolescents in Israel. Isr J Psychiatry. (2018) 55:22–30., PMID: 30351277

[56] Sanchez AL Comer JS LaRoche M . Enhancing the responsiveness of family-based CBT through culturally informed case conceptualization and treatment planning. Cognit Behav Pract. (2022) 29:750–70. doi: 10.1016/j.cbpra.2021.04.003

[57] Christon LM McLeod BD Jensen-Doss A . Evidence-based assessment meets evidence-based treatment: An approach to science-informed case conceptualization. Cognit Behav Pract. (2015) 22:36–48. doi: 10.1016/j.cbpra.2013.12.004

[58] Meld ST . Nasjonal helse- og sykehusplan (2020-2023) [National Health and Hospital Plan (2020-2023)] (white paper). Oslo: Ministry of Health and Care Services (2019).

[59] Lovdata . Lov om pasient- og brukerrettigheter (The law of patient - and user rights) [The law of patient - and user rights] (1999). Oslo: Helse- og omsorgsdepartementet. Available online at: https://lovdata.no/dokument/NL/lov/1999-07-02-63 (Accessed December 15, 2025).

[60] Helsedirektoratet . Nasjonalt kvalitetsindikatorsystem (NKI) - årsrapport 2021 (2022). Oslo: Helsedirektoratet. Available online at: https://www.helsedirektoratet.no/rapporter/nasjonalt-kvalitetsindikatorsystem-nki-arsrapport-2021#referere (Accessed December 15, 2025).

[61] Opptrappingsplan for barn og unges psykiske helse (2019–2024). Oslo: Government's strategy for mental health (2019).

[62] Norwegian Ministry of Health and Care Services . Riksrevisjonens undersøkelse av psykiske helsetjenester Dokument 3:13 (2020–2021) [Government's strategy for mental health] (2020). Oslo: Ministry of Health and Care Services. Available online at: https://www.riksrevisjonen.no/globalassets/rapporter/no-2020-2021/psykiske-helsetjenester.pdf (Accessed December 15, 2025).

[63] Barneombudet . Hvem skal jeg snakke med nå? Psykisk helsehjelp til barn og unge i kommunene (2022). Oslo: Barneombudet. Available online at: https://www.barneombudet.no/uploads/documents/Publikasjoner/Fagrapporter/Hvem-skal-jeg-snakke-med-na.pdf (Accessed December 15, 2025).

[64] Statens undersøkelseskommisjon for helse- og omsorgstjenesten UKOM . Ungdom med uavklart tilstand - Samhandling mellom kommunale tjenester og mellom kommunale tjenester og BUP. Oslo: UKOM (2020).

[65] Barneombudet . Jeg skulle hatt BUP i en koffert, en psykisk helsetjeneste tilpasset barn og unges behov (2020). Oslo: Barneombudet. Available online at: https://www.barneombudet.no/vart-arbeid/publikasjoner/jeg-skulle-hatt-bup-i-en-koffert (Accessed December 15, 2025).

[66] Migrasjonshelse (FHI) og Nasjonal kompetansetjeneste for samtidig rusmisbruk og psykisk lidelse (NKROP) . Kulturformuleringsintervjuet (KFI), DSM-5 Et klinisk verktøy i personsentrert og kultursensitiv kommunikasjon (2015). Oslo. Available online at: https://www.sykehuset-innlandet.no/4aea83/siteassets/fag-og-forskning/forskning-og-innovasjon/forskningssenter-for-eksistensiell-helse/kulturformuleringsintervjuet.pdf (Accessed December 15, 2025).

[67] Helsedirektoratet . Nasjonalt pasientforløp. Psykiske lidelser-barn og unge (2018). Oslo: Helsedirektoratet. Available online at: https://www.helsedirektoratet.no/nasjonale-forlop/psykiske-lidelser-barn-og-unge (Accessed December 15, 2025).

[68] Rousseau C Guzder J . Supplementary module 9: School-age children and adolescents. In: Lewis-Fernández R Aggarwal NK Hinton L Hinton DE Kirmayer LJ , editors. DSM-5® Handbook on the Cultural Formulation Interview. Washington DC: American Psychiatric Association Publishing (2016). p. 156–64.

[69] Helsedirektoratet . Internasjonalt perspektiv på psykisk helse og helsetjenester til mennesker med psykiske lidelser (2015). Norway Oslo: Helsedirektoratet. Available online at: https://www.helsedirektoratet.no/rapporter (Accessed December 15, 2025).

[70] Nasjonal kompetansetjeneste ROP . Hvordan bruke kulturformuleringsintervjuet (2016). Available online at: https://www.sykehuset-innlandet.no/fag-og-forskning/forskning/forskningssenter-for-eksistensiell-helse/kfi/kfi-i-klinisk-bruk/videoer/#hvordan-bruke-kfi-klinisk-eksempel (Accessed December 15, 2025).

[71] Aggarwal NK Glass A Tirado A Boiler M Nicasio A Alegría M . The development of the DSM-5 cultural formulation interview-fidelity instrument (CFI-FI): a pilot study. J Health Care Poor Underserved. (2014) 25:1397–417. doi: 10.1353/hpu.2014.0132, PMID: 25130248 PMC4306341

[72] Elo S Kyngäs H . The qualitative content analysis process. J Adv Nurs. (2008) 62:107–15. doi: 10.1111/j.1365-2648.2007.04569.x, PMID: 18352969

[73] Svamo NTØ Haug SHK DeMarinis V . I need to get back to a normal life": using the core DSM-5 Cultural Formulation Interview to explore existential themes shared by adolescents in specialized mental healthcare in Norway. Front Psychol. (2025) 16:1652189. doi: 10.3389/fpsyg.2025.1652189, PMID: 41312262 PMC12646933

[74] Paralikar VP Sarmukaddam SB Patil KV Nulkar AD Weiss MG . Clinical value of the cultural formulation interview in Pune, India. Indian J Psychiatry. (2015) 57:59–67. doi: 10.4103/0019-5545.148524, PMID: 25657458 PMC4314918

