# Correction: Efficacy pilot study of the DSM-5 Cultural Formulation Interview in a specialized mental healthcare inpatient unit for adolescents in Norway

**DOI:** 10.3389/fpsyt.2026.1789694

**Published:** 2026-01-26

**Authors:** Nina Therese Øversveen Svamo, Valerie DeMarinis, Sigrid Helene Kjørven Haug

**Affiliations:** 1 Research Center for Existential Health, Innlandet Hospital Trust, Faculty of Social and Health Sciences, University of Inland Norway, Lillehammer, Norway; 2 Research Center for Existential Health, Innlandet Hospital Trust, Brumunddal, Norway; 3 Department of Public Health and Clinical Medicine, Umeå University, Umeå, Västerbotten, Sweden; 4 Research Center for Existential Health, Innlandet Hospital Trust, Faculty of Social and Health Sciences, University of Inland Norway, Elverum, Norway

**Keywords:** adolescents, clinicians, communication, Cultural Formulation Interview, person-centered care, psychiatry

Affiliation for co-author Valerie DeMarinis was incorrectly given as:

Department of Public Health and Clinical Medicine, Research Center for Existential Health, Innlandet Hospital Trust, Umeå University, Umeå, Sweden.

The correct affiliations for Valerie DeMarinis are:

“Research Center for Existential Health, Innlandet Hospital Trust, Brumunddal, Norway” and “Department of Public Health and Clinical Medicine, Umeå University, Umeå, Västerbotten, Sweden”.

The original article has been updated.

