# Supplementary material for: Efficacy pilot study of the DSM-5 Cultural Formulation Interview in a specialized mental healthcare inpatient unit for adolescents in Norway
Source: Front Psychiatry. 2026 Jan 2;16:1595131. doi: 10.3389/fpsyt.2025.1595131 (PMC12807972; doi:10.3389/fpsyt.2025.1595131)
Supplement: Supplementary file 1 [file DataSheet1.pdf]

Supplementary material 1, Semi-structured interview for clinicians

Semi-structured, debriefing interview questions on feasibility, acceptability, and clinical utility for clinicians inspired by Aggarwal and colleagues' study (1). Questions 1, 3, 4, 5 and 6 are from the study and question 2 is partially reformulated (1). Questions 7-14 have been added by the current researcher (Valerie DeMarinis, Research Center for Existential Health, Innlandet Hospital Trust, Ottestad, Norway; Department of Public Health and Clinical Medicine, Umeå University, Sweden)

1. Overall, how would you describe your experience using these questions of the Cultural

Formulation Interview (CFI)?

2. How did the inclusion of the CFI affect the clinical treatment?

3. What impact did using the CFI have on your relationship with your patient?

4. How did the CFI affect the differential diagnosis and the eventual working diagnosis?

5. How did the CFI affect treatment planning?

6. What was most helpful about the inclusion of the CFI in the clinical evaluation? Least helpful?

7. Was it difficult to follow the structure of the interview from 1-16?

8. Was it difficult to motivate the patient or yourself during the interview?

9. Was the seriousness of their condition such that they were unable to answer the questions?

10. Was the interview difficult to carry out due to other agreements or treatment?

11. Was the CFI training you received relevant and useful?

12. Was there a connection between the patient's complaints and the basis and purpose of the CFI interview?

13. Is there a connection between the Innlandet Hospital Trust values (Quality and Knowledge, Openness and Involvement, Respect and Predictability) and the basis and purpose of the CFI interview? Elaborate on the way in which there is a connection/possibly no connection/ other notation.

14. Do you experience any difference between adolescents who receive CFI questions in assessment or treatment compared to those who have not received CFI questions? If yes, please describe.

1. Aggarwal NK, DeSilva R, Nicasio AV, Boiler M, Lewis-Fernández R. Does the Cultural Formulation Interview for the Fifth Revision of the Diagnostic and Statistical Manual of Mental Disorders (Dsm-5) Affect Medical Communication? A Qualitative Exploratory Study from the New York Site. *Ethn Health* (2015) 20(1):1-28.
