# Supplementary material for: Efficacy pilot study of the DSM-5 Cultural Formulation Interview in a specialized mental healthcare inpatient unit for adolescents in Norway
Source: Front Psychiatry. 2026 Jan 2;16:1595131. doi: 10.3389/fpsyt.2025.1595131 (PMC12807972; doi:10.3389/fpsyt.2025.1595131)
Supplement: Supplementary file 2 [file DataSheet2.pdf]

Supplementary material 2, T2 and T3 Semi-structured interviews for patients

The T2 interview is inspired by the Semi-structured, Debriefing Interview Questions on Feasibility, Acceptability, and Clinical Utility by Aggarwal and colleagues (1). The questions have been modified for clinical populations in Norway by the second author (Valerie DeMarinis, Research Center for Existential Health, Innlandet Hospital Trust, Ottestad, Norway; Department of Public Health and Clinical Medicine, Umeå University, Sweden).

The T3 questions were developed by researchers (Sigrid Helene Kjørven Haug, Research Center for Existential Health, Innlandet Hospital Trust, Ottestad, Norway; Faculty of Social and Health Sciences**,** University of Inland Norway, Elverum, Norway and Valerie DeMarinis, Research Center for Existential Health, Innlandet Hospital Trust, Ottestad, Norway; Department of Public Health and Clinical Medicine, Umeå University, Sweden) and experienced consultants.

T2 interview for patients:

1. How do you feel about being in this inpatient unit? Both positive and negative things.

2. How do you find communication with the people who work here?

3. It may be difficult to remember things from the CFI with the clinician, but is there anything you remember? How do you find communication with the clinicians?

(Here I may highlight some central themes or difficulties from the CFI in order to remind them and to see if they elaborate on these).

4. Do you think that your opinions and experiences are taken into account? In what way?

5. How did you feel about having CFI via video conference? (if applicable)

T3 interview for patients:

1. What is your experience of being in this inpatient unit? What are you satisfied with and what could be better?

2. Have you given any thought to the timing of the CFI, could it have come later in the treatment period?

3. What have you found most important during your stay here?

(Topics from the CFI interview can be included here if relevant)

4. How have you found communication with the people who work here?

(Topics from the CFI can also be included here if relevant)

5. How has it been that the clinician who conducted the CFI has not been in the inpatient unit that much? (If applicable)

6. Have you found that the team has used information from the CFI in the treatment? In what ways?

7. Have your opinions and experiences been taken into account? In what ways?

8. How do you think your life will be after this treatment period?

9. How has it been that parts of the treatment have taken place via video conference? (If applicable)

1. Aggarwal NK, DeSilva R, Nicasio AV, Boiler M, Lewis-Fernández R. Does the Cultural Formulation Interview for the Fifth Revision of the Diagnostic and Statistical Manual of Mental Disorders (Dsm-5) Affect Medical Communication? A Qualitative Exploratory Study from the New York Site. *Ethn Health* (2015) 20(1):1-28.
